# Complement-Activating Capacity of Autoantibodies Correlates With Disease Activity in Bullous Pemphigoid Patients

**DOI:** 10.3389/fimmu.2018.02687

**Published:** 2018-11-20

**Authors:** Roxana M. Chiorean, Adrian Baican, Mayson B. Mustafa, Annette Lischka, Daniel-Corneliu Leucuta, Vasile Feldrihan, Michael Hertl, Cassian Sitaru

**Affiliations:** ^1^Department of Dermatology, Medical Faculty, Medical Center - University of Freiburg, Freiburg, Germany; ^2^Department of Dermatology, University of Medicine and Pharmacy Iuliu Hatieganu, Cluj-Napoca, Romania; ^3^Department of Medical Informatics and Biostatistics, University of Medicine and Pharmacy Iuliu Hatieganu, Cluj-Napoca, Romania; ^4^Department of Immunology, University of Medicine and Pharmacy Iuliu Hatieganu, Cluj-Napoca, Romania; ^5^Department of Dermatology and Allergology, Philipps-University, Marburg, Germany; ^6^Centre for Biological Signaling Studies(BIOSS), University of Freiburg, Freiburg, Germany

**Keywords:** bullous pemphigoid, complement-activating capacity of autoantibodies, complement-binding test, disease activity, ABSIS, BPDAI

## Abstract

**Background:** Bullous pemphigoid is a subepidermal blistering skin disease, associated with autoantibodies to hemidesmosomal proteins, complement activation at the dermal-epidermal junction, and dermal granulocyte infiltration. Clinical and experimental laboratory findings support conflicting hypotheses regarding the role of complement activation for the skin blistering induced by pemphigoid autoantibodies. In-depth studies on the pathogenic relevance of autoimmune complement activation in patients are largely lacking. Therefore, the aim of this study was to investigate the pathogenic relevance of complement activation in patients with bullous pemphigoid. Complement activation by autoantibodies *in vivo* as measured by the intensity of complement C3 deposits in the patients' skin and *ex vivo* by the complement-fixation assay in serum was correlated with the clinical disease activity, evaluated by Autoimmune Bullous Skin Disorder Intensity Score (ABSIS) and Bullous Pemphigoid Disease Area Index (BPDAI), as well as, with further immunopathological findings in patients with bullous pemphigoid.

**Results:** Complement-activation capacity of autoantibodies *ex vivo*, but not deposition of complement in the perilesional skin of patients, correlates with the extent of skin disease (measured by ABSIS and BPDAI) and with levels of autoantibodies.

**Conclusions:** Our study provides for the first time evidence in patients for a pathogenic role of complement activation in bullous pemphigoid and should greatly facilitate the development of novel diagnostic tools and of more specific therapies for complement-dependent autoimmune injury.

## Background

Autoantibodies induce tissue damage in a series of autoimmune diseases, including rheumatoid arthritis, Goodpasture syndrome, myasthenia gravis, and autoimmune bullous dermatoses (1–8). Demonstration of the pathogenic potential of autoantibodies in these diseases greatly facilitated the development of effective B cell-targeted therapies (9). Binding of pathogenic autoantibodies to their targets induces tissue damage by various mechanisms. Autoantibodies may exert a direct effect just by binding to their target mediated by steric hindrance and/or by triggering the transduction of a signal to the cell. In other conditions, in addition to binding to their target antigen, autoantibodies need to interact with factors of the innate immune system, including the complement system and inflammatory cells in order to unfold their pathogenic potential (10). In patients with autoantibody-mediated diseases, deposits of complement components are frequently found in the affected tissues as an immunopathological marker of an inappropriate local complement activation (11, 12). Beyond their diagnostic significance, these clinical laboratory observations suggest a pathogenic role for complement activation in autoimmune diseases (9, 12).

The pathogenic significance of the complement activation in the setting of autoimmune injury has been extensively addressed using different *ex vivo* and animal models of autoimmune diseases (13–21). The results of these studies provide ample information suggesting that, in several conditions, including rheumatoid arthritis, myasthenia gravis, and epidermolysis bullosa acquisita, autoantibodies unfold their pathogenic potential by activating the complement cascade (13, 15, 17). In other diseases such as pemphigus vulgaris or anti-laminin 332 pemphigoid, complement activation by autoantibodies appears to be an epiphenomenon and is not required for tissue injury (16, 22–24).However, studies using experimental models of pemphigoid diseases provided partly conflicting results on the role of complement activation (14, 18–20). A new and interesting potential therapeutic target is represented by the C5 fraction of complement. While C5aR1 showed a proinflammatory effect in a mouse model of bullous pemphigoid and epidermolysis bullosa acquisita, C5aR2 demonstrated an anti-inflammatory effect in the same mouse model of bullous pemphigoid (21, 25). Understanding the role and mechanisms of complement activation in autoimmune diseases provides a basis for developing more specific diagnostic assays and therapeutic approaches targeting key complement components and pathogenic events.

Bullous pemphigoid (BP) is a prototypical organ-specific autoimmune dermatosis associated with complement activation (26). Deposits of several complement components, of which C3 deposition is of significance for the routine diagnosis, are characteristically found in patients with bullous and gestational pemphigoid (27, 28). These findings are matched by observations in several animal models of bullous pemphigoid induced by the passive transfer of antibodies against BP180 demonstrating deposits of complement C3 at the dermal-epidermal junction of the diseased mice or hamsters (18, 19, 29). The pathogenic significance of the complement activation has been addressed *in vivo* using mice deficient for several complement components and pharmacological inhibition of complement activation using cobra venom factor (14, 18–20, 30, 31). In several earlier studies, activation of complement was shown to be a prerequisite for blister induction by autoantibodies. However, intriguing results of more recent studies challenged the major pathogenetic role of the complement system in pemphigoid diseases. Specifically, skin fragility was also demonstrated to be induced in a complement-independent manner. Collagen XVII was depleted in cultured normal human keratinocytes using antigen-specific rabbit polyclonal IgG. Also, skin fragility was induced in neonatal collagen XVII-humanized mice by passive transfer of F(ab′)2 fragments of IgG autoantibodies against collagen XVII from bullous pemphigoid patients or rabbit (19). Furthermore, passive transfer of autoantibodies from bullous pemphigoid patients was shown to induce blister formation in neonatal C3-deficient collagen XVII-humanized mice (20). A possible mechanism of depletion is represented by binding of IgG autoantibodies and internalization from the cell membrane (32).

**Figure 1:**
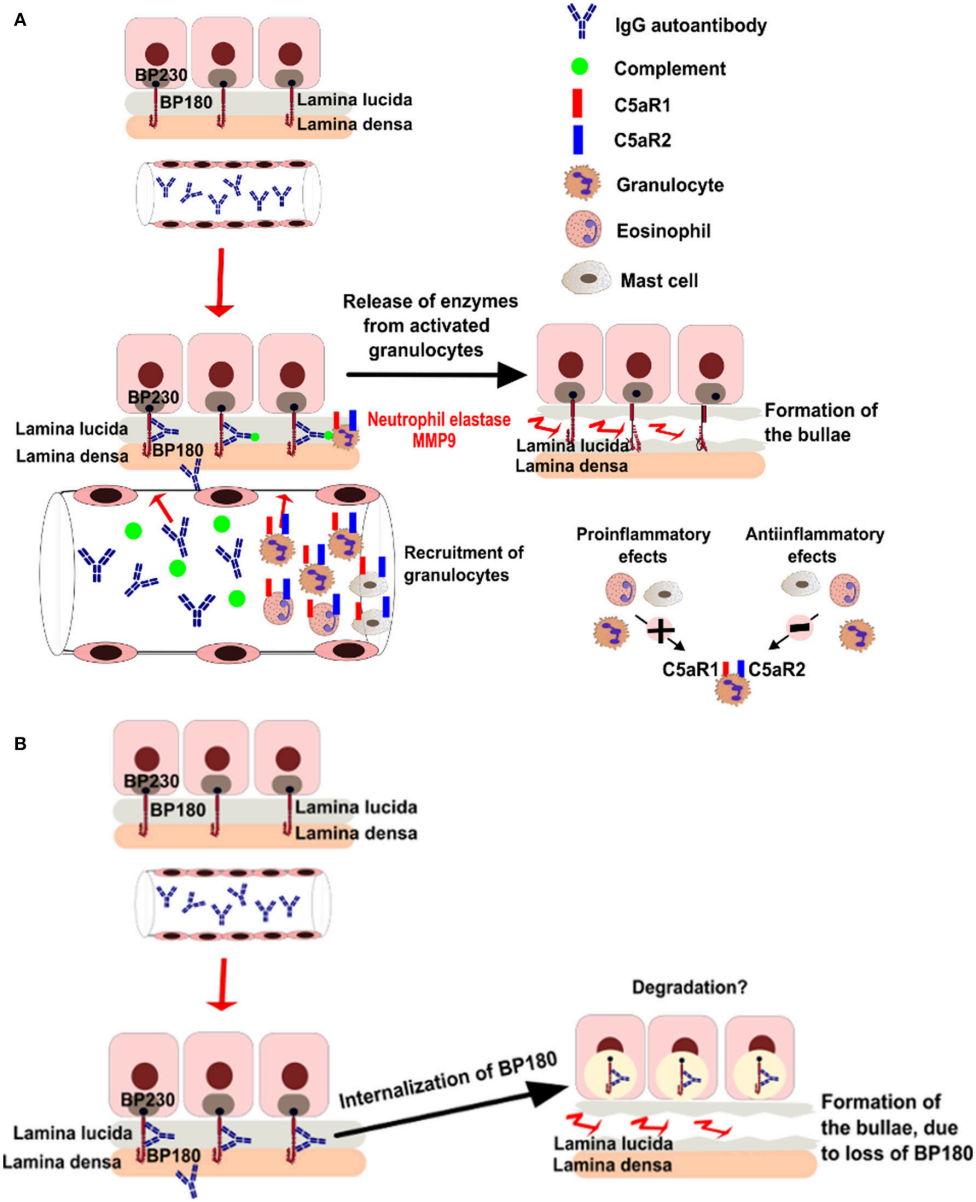
Putative pathogenic mechanisms of blistering in bullous pemphigoid. **(A)** Complement dependent pathways of bullae formation. Binding of IgG autoantibodies at the dermal epidermal junction leads to complement activation and recruitment of neutrophils. Activated neutrophils release proteolytic enzymes, inducing dermal-epidermal separation. **(B)** Complement-independent pathways of bullae formation. Binding of IgG autoantibodies to BP180 leads to depletion of the protein by internalization.

Additional studies using *ex vivo* models of pemphigoid diseases revealed complex and partly conflicting results (32–34), summarized in Figure 1. While these conflicting data may reflect different pathogenetic mechanisms of the autoantibody-induced tissue injury in various models, controversy surrounds the pathogenic relevance of complement activation in patients.

Therefore, in the present study, we aimed to investigate the pathogenic significance of complement activation in pemphigoid patients. For this purpose, we have measured the complement-binding capacity of patients' autoantibodies *in vivo* and *ex vivo* and have assessed their correlation with the clinical disease activity, as well as, their capacity to induce dermal-epidermal separation *ex vivo*. The results of our study documented a correlation of the autoantibody complement-binding capacity with the extent of the blistering disease, strongly suggesting a pathogenic role of complement activation in pemphigoid patients.

## Methods

### Patients

We have retrospectively analyzed 90 patients with BP, from the Department of Dermatology, at the University of Medicine and Pharmacy “Iuliu Hatieganu,” Cluj-Napoca, Romania. The study was approved by the local Ethic Committee and performed in accordance with the Declaration of Helsinki. Written informed consent was obtained from all patients, in adherence to the Helsinki Principles. Inclusion criteria for patients with BP were: (1) presence of skin bullae, (2) subepidermal blisters on histopathological examination and (3) linear deposits of IgG and/or C3 at the dermo-epidermal junction, detected by direct immunofluorescence. In addition, we have also evaluated the presence of anti-BP180 +/- anti-BP230 autoantibodies, measured by the enzyme-linked immunosorbent assay (ELISA) method and titers of IgG autoantibodies binding to the epidermal side, measured by indirect immunofluorescence (IF) microscopy on salt-split skin (4).

Epidemiological, clinical and immunological data were collected from patients' medical records. In addition to the age of onset and gender, we collected further variables, including erythrocyte sedimentation rate (ESR) at diagnosis and history of neurological comorbidities (stroke, dementia, Parkinson, epilepsy). Female to male ratio was 1.19 (49:41). Eleven BP patients presented oral mucosa involvement. The objective cutaneous and mucosal components of Autoimmune Bullous Skin Disorder Intensity Score (ABSIS) and Bullous Pemphigoid Disease Area Index (BPDAI) scores have been evaluated using clinical pictures of BP patients (35, 36). ESR was assessed both as a continuous and dichotomial variable. For the dichotomial value, we used a cut-off of 30 mm/h, as this was proved to represent a risk factor for lethal outcome in patients with BP (37).

The assessed immunopathological parameters were the direct and indirect IF microscopy findings, complement-binding test results, extent of dermal-epidermal split and titers of anti-BP180 and anti-BP230 autoantibodies.

### Direct immunofluorescence

Sections from perilesional patients' skin were fixed for 10 min in acetone and then washed 2 times in phosphate buffered saline (PBS). In a further step, they were incubated with rabbit anti-human IgG-FITC (Dako) diluted 1:100 in PBS and rabbit-anti-human C3-FITC (Dako) diluted 1:100 in PBS. Sections were washed 2 times, 10 min, in PBS (pH 7.2). At the end, sections were mounted in 50% glycerol-PBS and analyzed by fluorescence microscopy. The sections were analyzed using an Olympus BX40 microscope and pictures from representative areas were acquired. The images were saved in.tiff or.jpeg format.

### Indirect immunofluorescence microscopy

**Figure 2:**
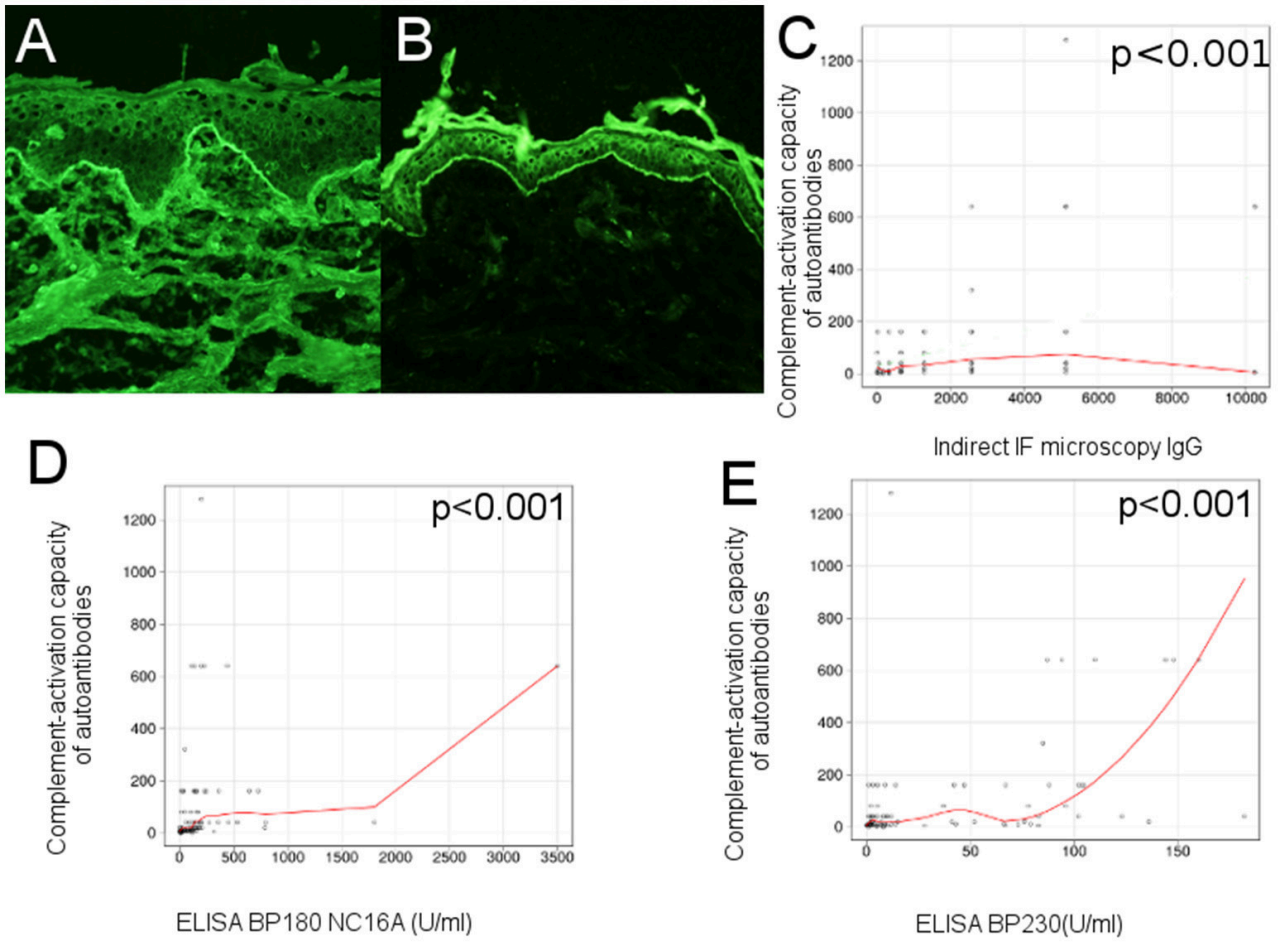
Complement-activation potential of circulating IgG anti-basement membrane zone autoantibodies correlates with their levels in bullous pemphigoid patients. **(A)** Indirect immunofluorescence microscopy on normal human foreskin shows binding of IgG autoantibodies from the serum of a BP patient at the dermal-epidermal junction. **(B)** Complement-binding test shows linear C3 deposits along the basement membrane of normal human foreskin, when using serum from a BP patient, demonstrating activation of complement by IgG autoantibodies. Relation of *ex vivo* complement-activating capacity of autoantibodies and **(C)**, titers of circulating IgG anti-basement membrane zone autoantibodies, measured by indirect IF microscopy; **(D)**, titers of circulating IgG anti-BP180 NC16A, measured by ELISA; **(E)**, titers of circulating IgG anti-BP230, measured by ELISA (*n* = 90). The red line represents the locally weighted scatterplot smoothing line.

**Figure 3:**
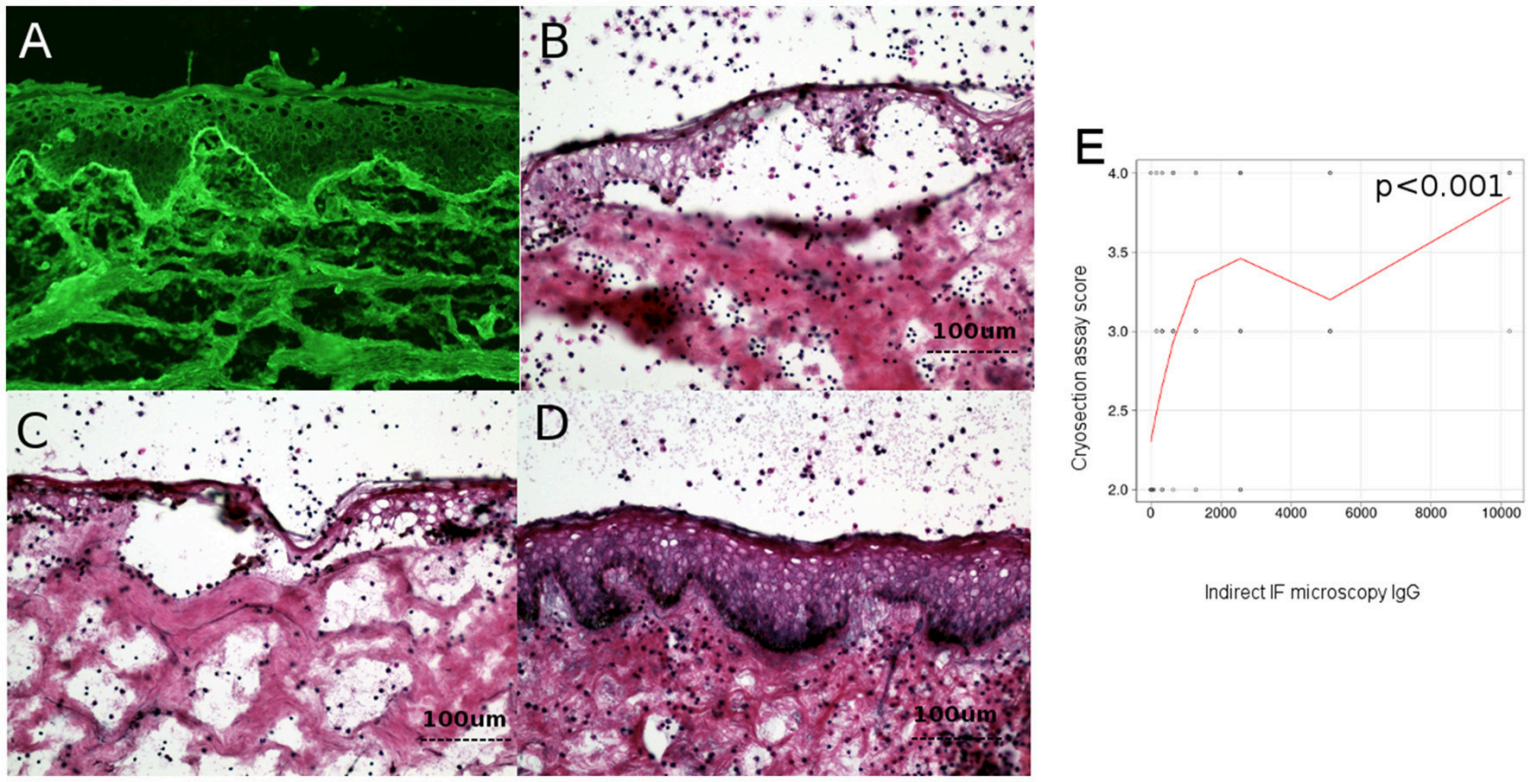
Level of circulating IgG anti-basement membrane zone autoantibodies correlates with dermo-epidermal separation in patients with bullous pemphigoid. **(A)** Indirect immunofluorescence microscopy on normal human foreskin, using BP patient sera. **(B,C)** Cryosection assay *ex vivo* of normal human foreskin, incubated with a BP serum pool **(B)** and BP serum from a patient **(C)**. Subsequent addition of normal human leukocytes leads to recruitment of neutrophils at the dermal-epidermal junction. Activation of neutrophils is induced by BP serum, leading to dermal-epidermal split. **(D)** Cryosection assay *ex vivo* of normal human foreskin, incubated with serum from healthy donors. Subsequent addition of normal human leukocytes does not lead to recruitment or activation of neutrophils at the dermal-epidermal junction and does not induce dermal-epidermal split. (**E)** Relation of titers of circulating IgG anti-basement membrane zone autoantibodies with dermal-epidermal separation in patients with bullous pemphigoid (*n* = 90). The red line represents the locally weighted scatterplot smoothing line.

Neonatal human foreskin, obtained from routine circumcision, was washed in cold PBS, cut into pieces of 6/15 μm, embedded in optimum cutting temperature compound and stored at −20°C until use. Sections were washed 2 times in PBS (pH 7.2), then incubated in a humid chamber, at room temperature, in two steps (1 h each). In the first step, the sections were incubated with serial dilutions of sera from BP patients and controls in 1% BSA PBS. The second step involved incubation with goat anti-human IgG (H + L), (AF488) diluted 1:100 in PBS (1% BSA). After each step, sections were washed 3 times, 10 min, in PBS (pH 7.2). At the end, sections were mounted in 50% glycerol-PBS and analyzed by fluorescence microscopy. An example is illustrated in Figures 2A, 3A. We have used the end-point titration of sera by indirect immunofluorescence microscopy as a robust indicator of autoantibody reactivity in patients with bullous pemphigoid.

### Measurement of the complement-activating capacity of autoantibodies *ex vivo* by complement-binding test method

The patient sera have been frozen at −80°C and then thawed and kept at 4C. The complement binding test was performed after the patients' sera were incubated at 56°C for 30 min to inactivate the complement components.

Neonatal human foreskin was prepared in the same manner as described above. Sections were washed 2 times in PBS (pH 7.2), then incubated in a humid chamber, at room temperature, in three steps (1 h each). In the first step, the sections were incubated with serial dilutions of sera from BP patients and controls, in PBS. In a second step, incubation was made with normal human fresh serum, as a source of complement (stored at −80°C before use), diluted at 1:5 in gelatin veronal buffer (Gelatin Veronal Buffer, BostonBio, Fisher Scientifics; Cat.no: IBB-290). The third step involved incubation with goat antiserum to human C3 (Polyclonal goat antibody to human C3-FITC, MPBiomedicals), diluted 1:100 in PBS (pH 7.2). After each step, sections were washed 3 times, 10 min, in PBS (pH 7.2). At the end, sections were mounted in 50% glycerol-PBS and analyzed by fluorescence microscopy.

An example is illustrated in Figure 2B.

### Detection of BP180- and BP230-specific autoantibodies

Levels of autoantibodies against the hemidesmosomal antigens BP180 and BP230 were measured using commercially available immunoassays (MBL laboratories, Nagoya, Japan), according to the manufacturer's instructions (38, 39).

### *Ex vivo* model of antibody-induced granulocyte-dependent dermal-epidermal separation

The ability of BP patients' autoantibodies to activate human leukocytes was evaluated using an *ex vivo* assay of antibody-induced granulocyte-dependent dermal-epidermal separation in cryosections of normal human skin, as described previously (34). Each experiment using the cryosection assay was performed twice to insure reproducibility of the results. The extent of dermal-epidermal separation (split) was assessed by two different observers and scored on a scale from 1 to 4, as follows: level 0-no split; level 1-split present in <25% of the basement membrane, but in more than 3 distinct places; level 2-split between 25 and 50% of the basement membrane; level 3-split between 50 and 75% of the basement membrane; level 4-split on >75% of the basement membrane.

Examples of cryosection assay are illustrated in Figures 3B–D.

### Measurement of complement activation in patients' skin

Direct IF microscopy pictures were taken with a 20x objective, using a Olympus BX40 microscope. The fluorescence intensity was assessed by two independent blinded observers using Image J software, version 1.47 v. The fluorescence intensity at the basement membrane was analyzed by choosing 50 points (using Multipoint selections tool), while background fluorescence was evaluated by selecting the whole dermis area, (using Freehand selections tool), as shown in detail in Figure 7. As we have seen a moderate agreement of intra- and inter- observer measurements, we have decided to further perform statistical analysis, using the results obtained after evaluation of one picture for each patient. A mean value of the fluorescence intensities was calculated as follows: fluorescence at the basement membrane mean = (fluorescence at the basement membrane observer 1 + fluorescence at the basement membrane observer 2)/2; Fluorescence of the background mean = (Fluorescence of the background observer 1 + fluorescence of the background observer 2)/2. Then, the following values were calculated: fluorescence difference = (fluorescence at the basement membrane mean–fluorescence of the background mean) and fluorescence ratio = (fluorescence at the basement membrane mean/fluorescence of the background mean). Further, we have performed a correlation of fluorescence difference and fluorescence ratio with the complement-activation capacity by autoantibodies.

### Statistical analysis

Association between continuous variables was assessed using the Spearman correlation. The Mann Whitney U test, medians and interquartile range were used for non-normally distributed variables. The relation between dichotomial variables was assessed using contingency tables with absolute values and percentages and Fisher's exact test. For all tests, a two-tailed *p*-value < 0.05 was considered statistically significant. Statistical analysis was performed using the R software environment for statistical computing and graphics, version 2.12.1 (copyright R Development Core Team, 2010) (40).

## Results

In order to compute the sample size for one of the main objectives of the study we considered as target effect size a minimum interesting Spearman correlation coefficient value of 0.3. For the simulation we considered the use of an exact test for Spearman correlation coefficient, with 80% power to detect the effect size, a 0.05 level of significance, and a two-tail *p*-value. The simulated sample size value obtained in these circumstances was 82 subjects (GPower 3.0.10 software, Germany).

### Complement-activation potential of circulating IgG anti-basement membrane zone autoantibodies correlates with their levels in bullous pemphigoid sera

In an initial evaluation of the complement-activating capacity of autoantibodies, we correlated the level of circulating IgG anti-basement membrane zone autoantibodies, measured by indirect IF microscopy and ELISA with titers of the complement-binding test. Complement activation correlated with levels of circulating IgG anti-basement membrane zone autoantibodies measured by indirect IF microscopy (*r* = 0.41 [95% CI 0.19–0.68], *p* < 0.001) (Figure 2C), level of anti-BP180 NC16A autoantibodies (*r* = 0.55 [95% CI 0.03–0.56], *p* < 0.001; Figure 2D) and level of anti-BP230 autoantibodies, (*r* = 0.46 [95% CI 0.17–0.7], *p* < 0.001; Figure 2E), both measured by ELISA.

We have observed a correlation between the level of circulating IgG autoantibodies, measured by indirect IF microscopy and the level of anti-BP180-NC16A autoantibodies (*r* = 0.22 [95% CI −0.02 to 0.34], *p* = 0.04) and BP230-specific autoantibodies (*r* = 0.33 [95% CI 0.25–0.64], *p* = 0.001), as measured by ELISA.

### Level of circulating IgG anti-basement membrane zone autoantibodies, but not complement activation by autoantibodies *ex vivo* correlates with dermo-epidermal separation in patients with bullous pemphigoid

To investigate the pathogenic relevance of complement activation *ex vivo*, measured by complement binding test, we have analyzed the correlation between the level of circulating IgG anti-basement membrane zone autoantibodies measured by indirect IF microscopy and ELISA and the complement-activation capacity of autoantibodies–as measured by the complement-binding test, with the extent of dermo-epidermal separation level in cryosection assay. Dermo-epidermal separation level in cryosection assay correlated with level of IgG autoantibodies measured by indirect IF (*r* = 0.4 [95% CI 0.2–0.5], *p* < 0.001) (Figure 3E), but not complement activation by autoantibodies, as measured by the complement-binding test (*p* = 0.09).

### Level of circulating BP180 NC16A-, but not BP230-specific autoantibodies correlates with disease activity in patients with bullous pemphigoid

**Figure 4:**
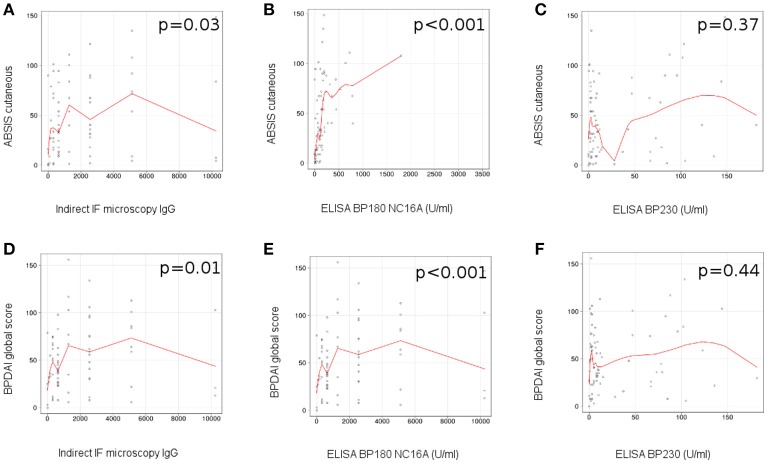
Level of circulating anti-BP180 NC16A, but not anti-BP230 autoantibodies, measured by ELISA correlates with disease activity in patients with bullous pemphigoid. Relation of titers of circulating IgG anti-basement membrane zone autoantibodies, measured by indirect IF microscopy with disease activity: **(A)**, ABSIS score of cutaneous involvement; **(D)**, BPDAI global score (*n* = 75). Relation of titers of circulating IgG anti-BP180 NC16A autoantibodies, measured by ELISA with disease activity: **(B)**, ABSIS score of cutaneous involvement; **(E)**, BPDAI global score (*n* = 75). Relation of titers of circulating IgG anti-BP230 autoantibodies, measured by ELISA with disease activity: **(C)**, ABSIS score of cutaneous involvement; **(F)**, BPDAI global score (*n* = 75). The red line represents the locally weighted scatterplot smoothing line.

We observed a correlation between the level of circulating IgG anti-basement membrane zone autoantibodies measured by indirect IF microscopy and the ABSIS score of cutaneous involvement (*r* = 0.25 [95% CI −0.06 to 0.51], *p* = 0.03; Figure 4A), BPDAI global score (*r* = 0.29 [95% CI −0.04 to 0.52], *p* = 0.01) (Figure 4D), BPDAI score of total skin involvement (*r* = 0.29 [95% CI 0–0.54], *p* = 0.01) and BPDAI score of blistering activity (*r* = 0.31 [95% CI −0.01 to 0.55], *p* = 0.006), but not BPDAI score of urticaria/erythema (*p* = 0.17).

We did not find a statistically significant association between the serum levels of IgG anti-basement membrane zone autoantibodies and the ABSIS objective oral score (*p* = 0.28) and BPDAI mucosal score (*p* = 0.28). Further, we did not observe a statistically significant difference between the levels of circulating IgG autoantibodies, measured by indirect IF microscopy, in the patients with associated mucosal involvement vs. patients with only cutaneous involvement (*p* = 0.32).

In patients with positive ELISA values, we observed a statistically significant correlation between the level of circulating BP180 NC16A-specific autoantibodies measured by ELISA and the ABSIS score of cutaneous involvement (*r* = 0.63 [95% CI 0.31–0.6], *p* < 0.001) (Figure 4B), BPDAI global score (*r* = 0.72 [95% CI 0.34–0.73], *p* < 0.001) (Figure 4E), BPDAI score of total skin involvement (*r* = 0.71 [95% CI 0.32–0.71], *p* < 0.001), BPDAI score of blistering activity (*r* = 0.63 [95% CI 0.27–0.69], *p* < 0.001) and the BPDAI score of urticaria/erythema (*r* = 0.69 [95% CI 0.22–0.59], *p* < 0.001), but not ABSIS objective oral score (*p* = 0.58) and BPDAI mucosal activity score (*p* = 0.72).

We did not observe a significant correlation between the level of circulating anti-BP230 autoantibodies measured by ELISA and the evaluated components of ABSIS and BPDAI scores (Figures 4C,F).

### *Ex vivo* complement-activation capacity of autoantibodies correlates with disease activity in patients with bullous pemphigoid

**Figure 5:**
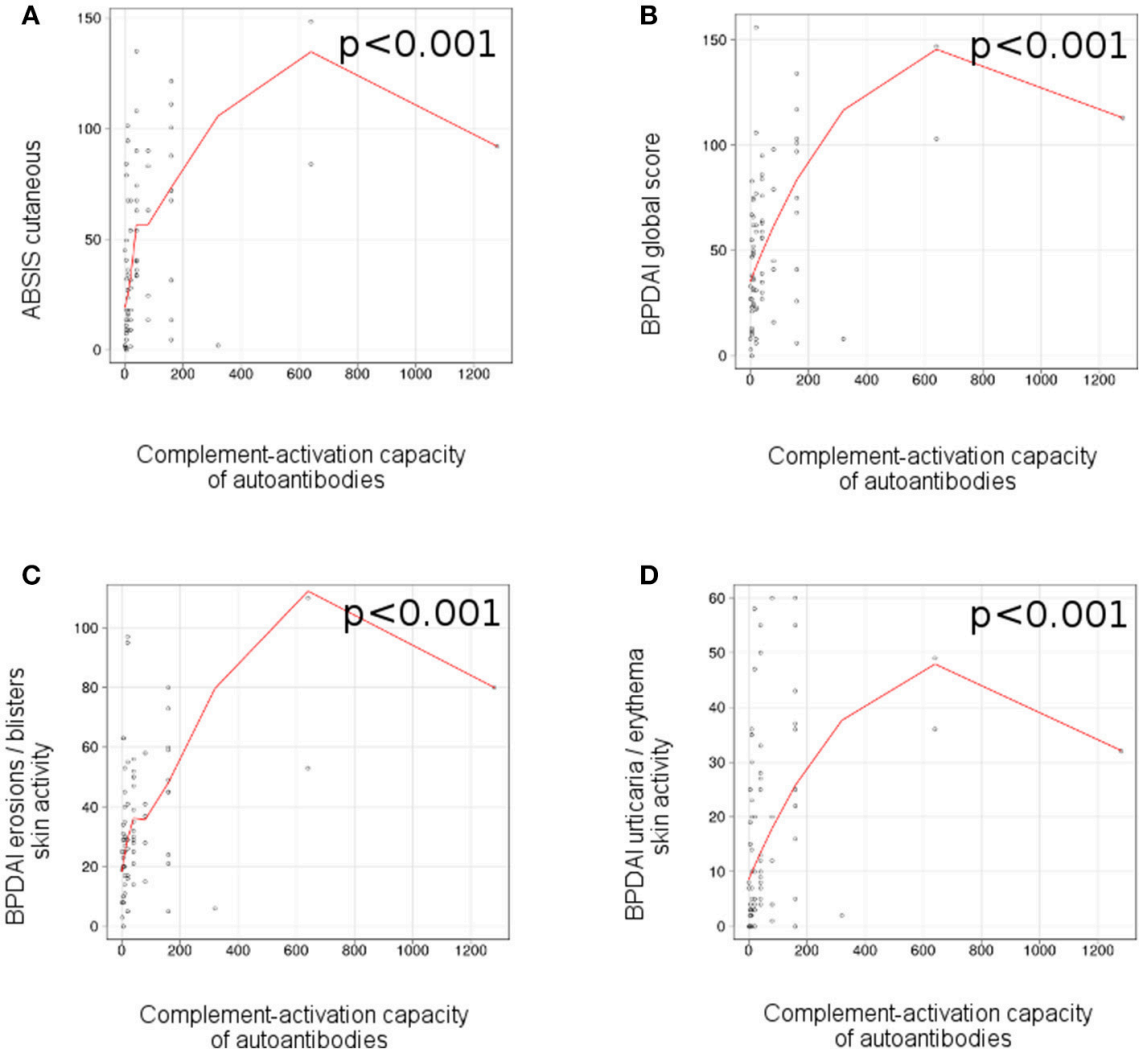
*Ex vivo* complement-activation capacity of autoantibodies correlates with disease activity in patients with bullous pemphigoid. Relation of *ex vivo* complement-activation capacity of autoantibodies with disease activity: **(A)**, ABSIS score of cutaneous involvement; **(B)**, BPDAI global score; **(C)**, BPDAI erosions / blisters skin activity; **(D)**, BPDAI urticaria/erythema skin activity (*n* = 75). The red line represents the locally weighted scatterplot smoothing line.

We observed a correlation between the complement-activation capacity of autoantibodies, measured by complement binding test and the ABSIS score of cutaneous involvement (*r* = 0.47 [95% CI 0.18–0.56], *p* < 0.001; Figure 5A), BPDAI global score (*r* = 0.47 [95% CI 0.16–0.59], *p* < 0.001; Figure 5B), BPDAI score of total skin involvement (*r* = 0.47 [95% CI 0.19–0.61], *p* < 0.001), BPDAI score of blistering activity (*r* = 0.41 [95% CI 0.09–0.62], *p* < 0.001; Figure 5C) and BPDAI score of urticaria/erythema (*r* = 0.45 [95% CI 0.15–0.47], *p* < 0.001; Figure 5D), but not ABSIS objective oral score (*p* = 0.8) and BPDAI mucosal activity score (*p* = 0.84). Patients with mucosal involvement showed slightly higher values of complement-activating capacity of autoantibodies, compared with patients with only cutaneous involvement (40 [7.5*-*120] vs. 20 [10*-*40]), but the results were not statistically significant (*p* = 0.76).

Autoantibodies in patients with positive ELISA findings also showed higher complement activation capacity, compared with patients with negative ELISA findings (40 [10-140] vs. 5 [4.25-6.25], *p* < 0.001).

Also, patients with positive ELISA values (for BP180-NC16A-, BP230-specific IgG autoantibodies or both) showed statistically significant higher values of ABSIS cutaneous score, BPDAI global score, BPDAI score of erosion activity and BPDAI score of urticaria/erythema, compared with patients with negative ELISA findings (Table 1).

**Table 1:** Values of different clinical and immunopathological variables according to ELISA positivity (^*^).

**Variable**	**ELISA positive**	**ELISA negative**	***p-*value**
Age (years)	(*n =* 82) 74.5 (68–80)	(*n =* 8) 68 (60.5–74.25)	0.35•
Indirect IF IgG	1,280 (400–2560)	640 (560–1600)	0.5•
Complement-binding test	40 (10–140)	5 (4.25–6.25)	< 0.001•
Cryosection assay score	3 (3–4)	3 (2.75–3)	0.44•
ABSIS cutaneous	(*n =* 68) 40.25 (17.62–72.56)	(*n =* 7) 9 (5.5–10)	0.01•
BPDAI global score	53.5 (30–76.25)	11 (8–29)	0.007•
BPDAI score of total skin involvement	49.5 (29.25–76.25)	11 (8–29)	0.009
BPDAI erosion activity	30 (20–49.25)	8 (6.5–26.5)	0.05•
BPDAI urticaria/ erythema	12.5 (4–32.25)	2 (0–3)	< 0.001•
Mucosal involvement	Yes (*n =* 11)	No (*n =* 0)	0.58+
ESR (mm/h)	(*n =* 66) 18.5 (11–32.75)	(*n =* 7) 15 (12.5–28)	0.72•

• *Mann-Whitney U test has been used*.

+ *Fisher Exact test has been used*.

* *ELISA positivity is defined as presence of a level of anti-BP180 NC16A autoantibodies and/or anti-BP230 autoantibodies ≥ 9 U/mL*.

### Dermo-epidermal separation measured by cryosection assay shows a weak correlation with disease activity in patients with bullous pemphigoid

We observed a weak correlation between the dermo-epidermal separation level, revealed by cryosection assay and the ABSIS score of cutaneous involvement (*r* = 0.24 [95% CI 0.02*-*0.44], *p* = 0.04), but not BPDAI global score, BPDAI score of total skin involvement, BPDAI score of blistering activity, BPDAI score of urticaria/erythema, ABSIS oral 1 score and BPDAI score of mucosal activity. We did not observe a statistically significant difference between the level of dermo-epidermal separation, revealed by cryosection assay in the patients with associated mucosal involvement vs. patients with only cutaneous involvement (*p* = 0.94).

### High pemphigoid disease activity associates with increased systemic inflammatory status

We did not find a statistically significant correlation between the ESR level taken as a continuous variable (mm/h) and the immunopathological features, as well as the evaluated components of ABSIS and BPDAI scores.

**Figure 6:**
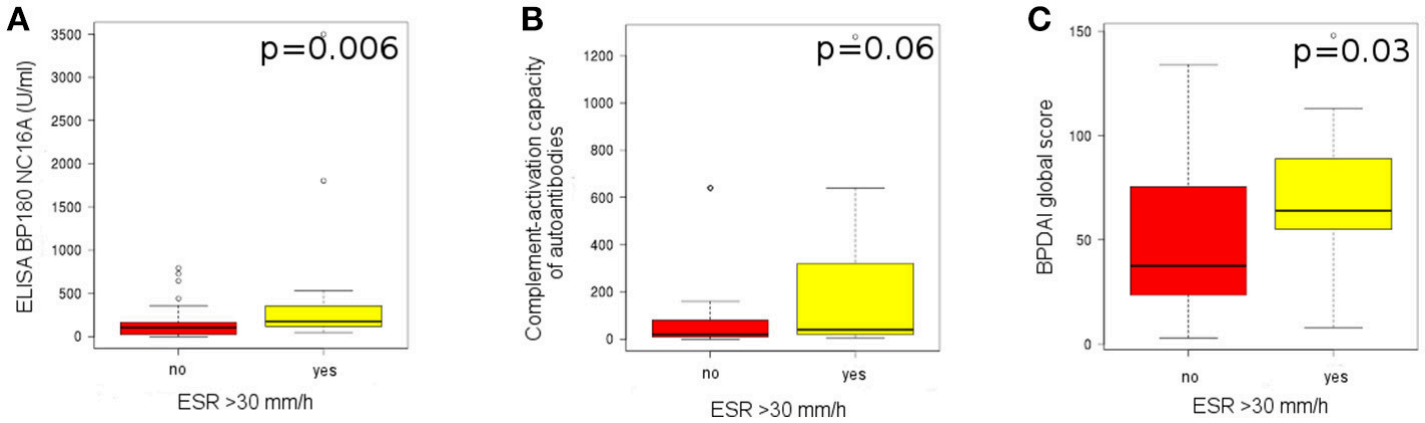
High pemphigoid disease activity associates with increased systemic inflammatory status. **(A)** Level of anti-BP180 autoantibodies, measured by ELISA, in patients with an ESR>30 mm/h (*n* = 18), compared with patients with an ESR<30 mm/h (*n* = 55). **(B)** Level of complement activation by IgG autoantibodies, measured by complement-binding test, in patients with an ESR>30 mm/h (*n* = 18), compared with patients with an ESR<30 mm/h (*n* = 55). **(C)** Level of BPDAI global score, in patients with an ESR>30 mm/h (*n* = 13), compared with patients with an ESR<30 mm/h (*n* = 48).

Patients with an ESR>30 mm/h showed a statistically significant higher level of anti-BP180 NC16A autoantibodies, measured by ELISA, compared to patients with an ESR<30 mm/h (174.1 [121.5*-*323.75] vs. 104.9 [25.25–163.3], *p* = 0.006) (Figure 6A). Also, they showed a higher level of complement activation (40 [20*-*280] vs. 20 [10–80]), but the results were not statistically significant (*p* = 0.06; Figure 6B).

**Figure 7:**
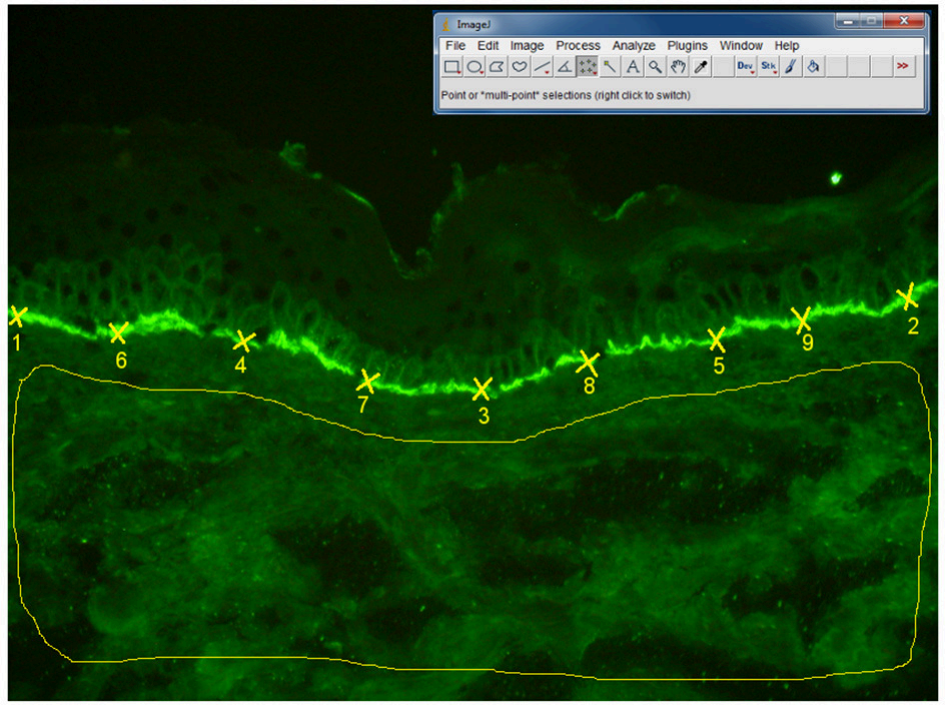
Algorithm of measurement of fluorescence intensity in direct IF microscopy images, in patients with BP (*n* = 27 patients). The fluorescence intensity was assessed by two independent blinded observers using Image J software, 1.47v version. The fluorescence intensity at the basement membrane was analyzed by selecting 50 points (using Multi-point tool), each point being situated at the half distance between two previous points. Background fluorescence was evaluated by selecting the whole dermis area, using “Freehand selections” tool. In order to obtain the mean value and standard deviation of the fluorescence intensity for the 50 points on the basement membrane and dermis background we have used the Analyze-Measure-Summarize tool.

Patients with an ESR>30 mm/h also showed a statistically significant higher BPDAI global score, compared with patients with an ESR < 30 mm/h (64 [55–89] vs. 37.5 [23.75-75.25], *p* = 0.03; Figure 6C) and a higher BPDAI skin erosion activity score (50 [34-56] vs. 27.5 [17-40.5], *p* = 0.005).

### Immunofluorescence intensity of C3 deposits in the skin of the patients does not correlate with complement-activating capacity of pemphigoid autoantibodies

We did not find a statistically significant correlation between the complement-activating capacity of autoantibodies, measured by complement binding test and the intensity of complement deposits in the skin, measured by our Image J algorithm of quantifying fluorescence (*n* = 27), for both Fluorescence difference (*r* = −0.07 [95% CI −0.47 to 0.29], *p* = 0.71) and Fluorescence ratio (*r* = −0.16 [95% CI −0.43 to 0.09], *p* = 0.43).

Also, we did not observe a correlation between the intensity of complement deposits in the skin and the immunopathological features of the patients.

Eighty-seven out of ninety patients showed a positive complement-binding test. The C3 deposits in DIF were evaluated by Image J in 27 patients. Out of these, 26 were able to induce complement-fixation *ex vivo*.

We did not find a statistically significant correlation between the complement-activating capacity of autoantibodies, measured by complement binding test and the intensity of complement deposits in the skin, measured by our Image J algorithm of quantifying fluorescence in patients with a positive complement-binding test (*n* = 26), for both Fluorescence difference (*r* = −0.21 (95% CI −0.51 to 0.25), *p* = 0.315) and Fluorescence ratio (*r* = −0.2 (95% CI −0.45 to 0.08), *p* = 0.316).

### Immunofluorescence intensity of C3 deposits in the skin of the patients does not correlate with disease activity in patients with bullous pemphigoid

To investigate the relation between the complement activation *in vivo* and disease activity, we correlated the intensity of complement deposits in the skin, measured by our Image J algorithm of quantifying fluorescence, for both fluorescence difference and fluorescence ratio with the evaluated components of ABSIS and BPDAI scores (*n* = 25). We did not find a statistically significant correlation between the complement activation *in vivo* and disease activity.

## Discussion

To investigate the pathogenic relevance of complement activation in pemphigoid patients, we have measured the complement-binding capacity of patients' autoantibodies *ex vivo* and in the patients' skin and have assessed their correlation with the clinical disease activity as well as their capacity to induce dermal-epidermal separation *ex vivo*. The salient findings of this study show for the first time that the complement-activating potential of autoantibodies *ex vivo* correlate well with both their serum levels and with pemphigoid disease activity in patients.

Based on the observation that complement deposits are constantly found, frequently to a higher extent when compared with IgG deposits at the dermal-epidermal junction in patients with bullous and gestational pemphigoid, it was early hypothesized that complement activation plays a major pathogenetic role for skin blistering in pemphigoid diseases (27, 28, 41, 42).

Previous studies have already shown that IgG4 and IgG1 are the predominant IgG subclasses present in the serum and binding to the dermoepidermal junction in patients with bullous pemphigoid. While a NC16A-specific IgG1 response was predominant in the acute phase of bullous pemphigoid, IgG4 was predominantly detected in bullous pemphigoid patients in remission. Using immunoafinity purified IgG subclasses from bullous pemphigoid patients, it has been demonstrated that IgG1, but not IgG4 autoantibodies activate the complement system *in vitro* (24).

Subsequent work using an assay in cryosections of human skin incubated with pemphigoid autoantibodies and granulocytes from healthy donors strengthened this concept by showing that complement inactivation results in less dermal-epidermal separation (33). However, further work using this *ex vivo* model of autoantibody-induced granulocyte-dependent tissue damage extended and partly corrected these findings. This suggested that, while recruitment of granulocytes heavily depends on complement activation by autoantibodies, none of these 2 events are strictly required for inducing the disease-specific effects in the cryosections, especially when granulocytes at high densities were used (34, 43). These findings may be explained by the design of cryosection assay. Since granulocytes in high numbers are directly placed on the sections at the dermal-epidermal junction, they do not have to migrate to the tissue-bound immune complexes. Therefore, chemotactic factors resulting from complement activation are not required for subepidermal split formation (34).

The central finding of our study that the complement-activating capacity of autoantibodies correlates well with the disease activity is in line with observations in animal models of pemphigoid diseases and the pemphigoid-like form of epidermolysis bullosa acquisita induced by the passive transfer of antibodies against the epidermal basement membrane (14, 17, 44). As expected, complement-activation by autoantibodies showed in general a good correlation with levels of autoantibodies as measured by their indirect IF microscopy titers. Autoantibodies in patients with bullous pemphigoid are mainly targeting the hemidesmosomal proteins collagen XVII/BP180 and BP230 (45, 46). Not surprisingly, the best correlation was documented with ELISA levels of BP180 NC16A-specific autoantibodies, which are believed to primarily mediate tissue damage in pemphigoid (29, 47–50). Data emerging from another group using mice deficient for murine collagen XVII and expressing the human ortholog of the hemidesmosomal antigen suggest that pemphigoid autoantibodies may induce tissue damage in both complement-dependent and complement-independent manner (19, 20, 31). However, the role of complement-independent mechanisms, including antigen depletion for the autoantibody-induced blistering in pemphigoid patients, especially in the generalized, inflammatory disease is still unclear (26). Integrating these novel findings in patients into the existing knowledge framework adds critical support for a pathogenic role of complement activation in the pemphigoid blister formation.

The *in vivo* deposition of complement at the dermal-epidermal junction in patients' skin as measured by the fluorescence intensity of C3 staining did not correlate with the complement-activating capacity of autoantibodies *ex vivo* or with the disease scores. The reason for this phenomenon is not known. Beyond issues related to an accurate measurement of tissue-bound complement deposits, one can only speculate that granulocyte recruitment shows a non-linear dependency on the production of complement-derived chemotactic factors. Indeed neutrophil activation in the upper dermis would also result in the production of chemotactic factors such as interleukin-8, which may amplify the recruitment of granulocytes and the extent of skin blistering. These findings parallel previous results suggesting that local complement activation correlates with tissue damage in a non-linear fashion, i.e., it needs to exceed a threshold in order to initiate a pathogenic inflammatory cascade eventually resulting in tissue damage (51).

Skin lesions in pemphigoid patients are usually associated with intense itch, which however has not yet been reliably reproduced in experimental models of pemphigoid diseases. Interestingly, more recent data show that mice carrying a deletion in the NC14A domain of murine type XVII collagen begin scratching early and develop erosions, subepidermal vesicles, eosinophil-rich skin infiltrates, and possibly autoantibodies (52). While not showing full-blown autoimmune blistering disease, these mice may indeed represent a first pemphigoid animal model featuring pruritus in immunocompetent mature animals (52, 53). While the pathogenesis of pruritus is complex (54), its mechanisms in pemphigoid diseases are still elusive. However, observations in patients suggest that pemphigoid itch may be related to the presence of IgE autoantibodies against the basement membrane with subsequent eosinophil and mast cell activation (55, 56). While a primary pathogenic involvement of complement activation in pruritus in pemphigoid appears unlikely and should be addressed in future studies on mouse models, the correlation of itch as measured by the subjective disease scores with the levels of complement-activation by autoantibodies may be addressed in a prospective future study.

A further finding of the present study is the correlation between markers of systemic inflammation and complement-activating capacity of autoantibodies. ESR is a robust marker of systemic inflammation, which however does not always correlate with the extend of complement activation as known from findings in patients with systemic lupus erythematosus. Patients with an ESR > 30 mm/h showed higher levels of BP180-specific autoantibodies and tendentially an increased level of complement activation by autoantibodies. These findings suggest that increased local inflammation “spills” systemically. Importantly, the present and previous findings show that an ESR>30 mm/h correlates with disease activity and is a risk factor for lethal outcome in patients with pemphigoid (37).

The association of bullous pemphigoid with neurological comorbidities is well characterized (57–59). Even if a major role of autoantibodies in the pathogenesis of neuronal tissue injury in pemphigoid diseases cannot be ruled out at this stage, data regarding an association between presence of neurological comorbidities and level of autoantibodies remains controversial (60, 61).

Our present results provide further rationale for the development of complement-targeted therapeutics in pemphigoid and diseases with related complement-dependent pathogenesis. Indeed inhibitors of terminal complement activation such as Eculizumab, which represented a major leap forward in the treatment of paroxysmal nocturnal hemoglobinuria could be repurposed for the treatment of pemphigoid diseases (62). Further complement inhibitors, including those targeting C3 activation that are currently under development may be also worth considering as therapeutic options in pemphigoid diseases (63).

Measuring the complement-activating autoantibodies may represent a significant improvement over existing diagnostic and monitoring tools in complement-mediated autoimmune diseases. It is known that binding of autoantibodies to their targets *per se* may not be sufficient to induce disease, which may require triggering Fc-dependent complement activation (64). Measuring specifically levels of complement-fixing autoantibodies by a quantitative complement-mediated assay may thus represent a diagnostic tool of pathogenic relevance, which may be especially useful for monitoring serological activity. In addition to the complement-fixation tests by indirect IF microscopy (28, 65), development of a quantitative immunoassay measuring complement activation by antigen-specific autoantibodies should be an important addition to the routine diagnosis of autoimmune diseases.

A limitation of the study is related to the way the evaluation of fluorescence intensity of C3 in direct fluorescence sections correlates with complement-activating capacity of autoantibodies.

An issue to be addressed in future studies is represented by the role of the eosinophils in the pathogenesis of bullous pemphigoid and possibly in complement activation. In a recent published study, it was shown that IL-5-activated eosinophils directly contribute to blister formation in the presence of bullous pemphigoid autoantibodies, in an *ex vivo* human disease model (66). In conclusion, the results of our study show that autoantibody complement-binding capacity correlates with the extent of the blistering disease, strongly suggesting a pathogenic role of complement activation in pemphigoid patients. These findings should greatly facilitate the development of more specific diagnostic and monitoring tools as well as the design of complement-targeted therapeutic approaches in autoimmune inflammatory diseases.

## Conclusions

In conclusion, complement activation by autoantibodies *ex vivo* as measured by the complement-fixation assay in serum was correlated with the clinical disease activity in patients with bullous pemphigoid. Our study provides for the first time evidence in patients for a pathogenic role of complement activation in bullous pemphigoid and should greatly facilitate the development of novel diagnostic tools and of more specific therapies for complement-dependent autoimmune injury.

## Data availability statement

The datasets during and/or analyzed during the current study are available from the corresponding author on reasonable request.

## Author contributions

RC and CS conceived of the study, made the design, coordinated the study, and drafted the manuscript. AB provided all patient data and biological samples. VF and RC collected the epidemiological data. RC, AL, and MM performed the laboratory experiments and interpretation if data. D-CL performed the statistical analysis. D-CL, RC, AL, and MM performed interpretation of the results. MH contributed to drafting and critical revision of the manuscript. All authors read and approved the final manuscript.

### Conflict of interest statement

The authors declare that the research was conducted in the absence of any commercial or financial relationships that could be construed as a potential conflict of interest.

## Abbreviations

ABSIS: Autoimmune Bullous Skin Disorder Intensity Score
BP: bullous pemphigoid
BPDAI: Bullous Pemphigoid Disease Area Index
BSA: bovine serum albumine
C3: complement component 3
ELISA: enzyme-linked immunosorbent assay
IF: immunofluorescence
IgG: immunoglobulin G
PBS: phosphate-buffered saline.
